# Crystal structure of di­chlorido­{2-methyl-2-[(pyridin-2-ylmeth­yl)amino]­propan-1-ol-κ^3^ *N*,*N*′,*O*}copper(II) from synchrotron data

**DOI:** 10.1107/S2056989016013773

**Published:** 2016-09-05

**Authors:** Jong Won Shin, Dong Won Lee, Dae-Woong Kim, Dohyun Moon

**Affiliations:** a Daegu-Gyeongbuk Branch, Korea Institute of Science and Technology Information, 90 Yutongdanji-ro, Buk-gu, Daegu 41515, Republic of Korea; b Beamline Department, Pohang Accelerator Laboratory, 80 Jigokro-127-beongil, Nam-Gu Pohang, Gyeongbuk 790-784, Republic of Korea

**Keywords:** crystal structure, hydrogen bond, π–π inter­actions, square-pyramidal geometry, synchrotron data

## Abstract

The title compound, [CuCl_2_(C_10_H_16_N_2_O)], has been synthesized and characterized by synchrotron single-crystal X-ray diffraction and FT–IR spectroscopy. The 2-methyl-2-[(pyridin-2-ylmeth­yl)amino]­propan-1-ol (mpmapOH) ligand, including pyridine, amine and hy­droxy groups, was synthesized by the reaction of 2-amino-2-methyl­propan-1-ol with pyridine-2-carbaldehyde and was characterized by NMR spectroscopy. In its Cu^II^ complex, the metal ion has a distorted square-pyramidal coordination geometry with two N and one O atom of the mpmapOH ligand and one chloride anion in the equatorial plane, and the second chloride in an axial position. The bond lengths involving the Cu^II^ ion range from 1.9881 (10) to 2.0409 (9) for the Cu—N and Cu—O bonds, and from 2.2448 (5) to 2.5014 (6) Å for the equatorial and axial Cu—Cl bonds, respectively. Inter­molecular hydrogen bonds (N—H⋯Cl and O—H⋯Cl) and face-to-face π–π inter­actions stabilize the mol­ecular structure and give rise to a two-dimensional supra­molecular structure extending parallel to (101).

## Abstract 2

The Cu^II^ ion in the title compound shows a distorted square-pyramidal coordination geometry with two N and one O atoms of the mpmapOH ligand and two Cl anions. In the crystal, mol­ecules are connected by hydrogen bonds and π–π inter­actions, forming a strong supra­molecular network along the *a*-axis direction.

## Chemical context   

Polyamine ligands have attracted much inter­est in the development of coordination and bio-inorganic chemistry because they can easily bind or inter­act with transition metal ions and form stable multifunctional metal complexes with significant potential applications in catalysis (Ahn *et al.*, 2016 ▸), magnetic materials (Benelli *et al.*, 2013 ▸) as well as pharmacology (Stringer *et al.*, 2015 ▸). For example, various platinum complexes including polyamine ligands or their derivatives have been synthesized and investigated as potential anti­cancer agents, *e. g*. nedaplatin, hepta­platin, and lobaplatin (Kapdi & Fairlamb, 2014 ▸). In particular, polyamine derivatives containing hydroxyl groups can easily form various multinuclear metal complexes and supra­molecular compounds because the hydroxyl groups can be fully or partially deprotonated and act as hydrogen-bonding donors and/or acceptors. For example, bpaeOH [bpaeOH = *N*,*N*-bis­(2-pyridinmeth­yl)-2-amino­ethanol] and H_2_pmide [H_2_pmide = *N*-(2-pyridyl­meth­yl)iminodi­ethanol] ligands containing pyridine, amine and hydroxyl groups have been used to form multinuclear iron(III) complexes (Shin *et al.*, 2014 ▸) and mixed-valence cobalt(II/III) complexes and have shown significant magnetic inter­actions and catalytic activities toward various olefins and alcohols (Shin *et al.*, 2011 ▸). Chloride ions in such complexes can easily bridge two metal ions, allowing the assembly of supra­molecular compounds (Sabounchei *et al.*, 2015 ▸).

Here, we report the synthesis and crystal structure of a copper(II) complex constructed from a versatile tridentate ligand, 2-methyl-2-[(2-pyridinylmeth­yl)amino]-1-propanol (mpmapOH; C_10_H_16_N_2_O), [Cu(mpmapOH)Cl_2_], (**I**).
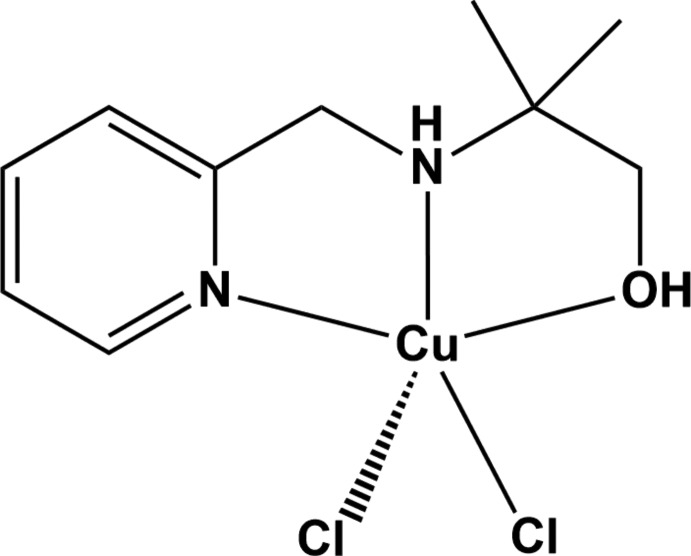


## Structural commentary   

**Figure 1:**
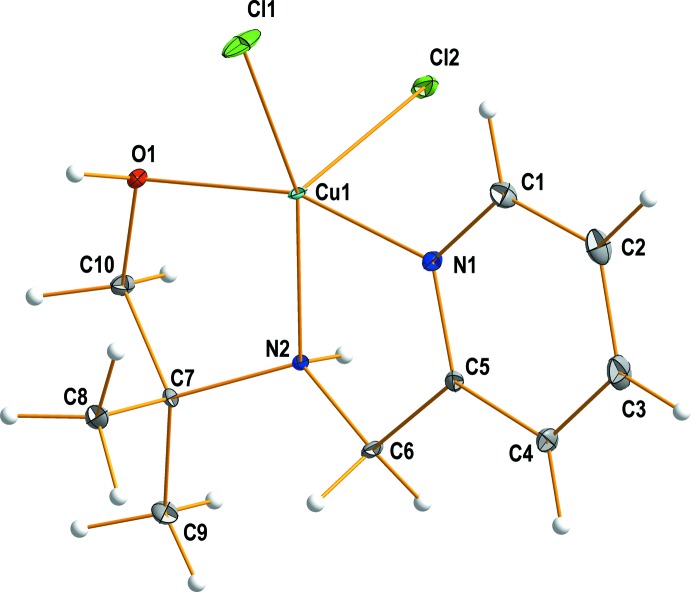
View of the mol­ecular structure of the title compound, showing the atom-labelling scheme, with displacement ellipsoids drawn at the 50% probability.

In the title compound (**I**) (Fig. 1 ▸), the copper(II) ion is five-coordinated by two nitro­gen and one oxygen atoms from the mpmapOH ligand and by two chloride anions. The coordination geometry around the copper ion can be described as distorted square-pyramidal. The equatorial plane consists of the two nitro­gen (N1 and N2) atoms and the hydroxyl group (O1) of the mpmapOH ligand and one chloride anion (Cl1). The coordination geometry is completed by an axial coordination of the second chloride anion (Cl2). The chloride anions coordinate in a *cis* position to each other. The Cu—*L*_mpmapOH_ bond lengths are in the range 1.9881 (10) to 2.0409 (9) Å. The Cu—Cl bond lengths are 2.2448 (5), and 2.5014 (6) Å, respectively, with the larger value corresponding to the axial chloride ligand. The equatorial atom Cl1 lies 0.332 (1) Å above the equatorial plane, away from the axial chloride anion Cl2. The bite angles of the five-membered chelate rings involving C5, C6 and C7, C10 atoms are 82.92 (4) and 82.97 (4)°, respectively. The bond angles around the copper ion range from 82.92 (4) to 161.51 (4)°.

## Supra­molecular features   

**Figure 2:**
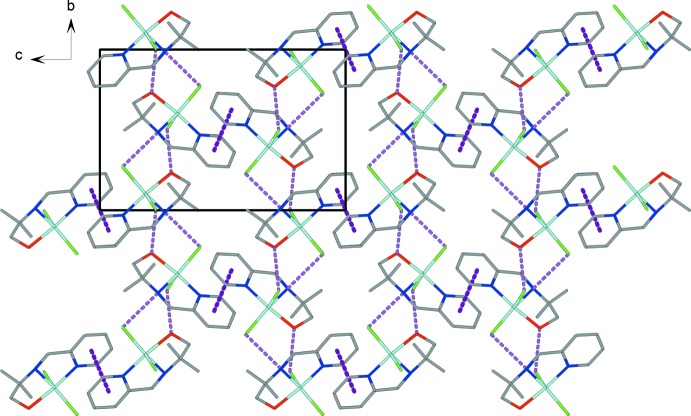
View of the crystal packing of the title compound, showing the N—H⋯Cl and O—H⋯Cl hydrogen bonds (pink dashed lines) and π–π inter­actions (purple dashed lines).

The two chloride anions form strong inter­molecular hydrogen bonds with secondary amine and hydroxyl groups of adjacent mpmapOH ligands, giving rise to a polymeric chain along the *b* axis (Fig. 2 ▸ and Table 1 ▸) (Steed & Atwood, 2009 ▸). The hydrogen-bonded polymeric chains are linked by face-to-face π–π inter­actions between the pyridine groups of the mpmapOH ligand with a centroid-to-centroid distance of 3.764 (1) Å and an inter­planar separation of 3.745 (1) Å. These inter­actions give rise to a two-dimensional supra­molecular network with layers parallel to (101) (Fig. 2 ▸).

## Database survey   

A search of the Cambridge Structural Database (Version 5.37, Feb 2016 with two updates; Groom *et al.*, 2016 ▸) did not show any related metal complexes with an mpmapOH ligand. The mpmapOH ligand was newly synthesized and the title compound is the first metal complex using mpampOH ligand for this research.

## Synthesis and crystallization   

The title compound (**I**) was prepared as follows. 2-Amino-2-methyl-1-propanol (4.90 g, 0.050 mol) was dissolved in MeOH (30 mL) followed by the addition of 2-pyridine­carboxaldehyde (5.41 g, 0.050 mol) under a nitro­gen atmosphere. The resulting mixture was strirred at room temperature for three hours, and then NaBH_4_ (6.05 g, 0.16 mol) was added slowly. The mixture was again stirred at room temperature overnight. The yellow solution was evaporated to dryness under reduced pressure. The residue was dissolved in CH_2_Cl_2_ and the undissolved solids were filtered off. The solution was washed with H_2_O and dried over MgSO_4_. After removal of the drying agent and solvent, the mpmapOH ligand was obtained as a yellow oil. Yield: 6.67 g (74%). ^1^H NMR (500 MHz, DMSO): *δ* = 0.98 (*s*, 6H, NH–C(*CH_3_*)_2_–CH_2_), 3.22 (*s*, 2H, NH–C(CH_3_)_2_–*CH_2_*–OH), 3.75 (*s*, 2H, Py–*CH_2_*–NH), 7.21 (*t*, 1H, 5.9 Hz, Py–*H*), 7.42 (*d*, 1H, 7.8 Hz, Py–*H*), 7.71 (*t*, 1H, 7.65 Hz, Py–*H*), 8.45 (*d*, 1H, 4.75 Hz, Py–*H*). To an MeOH solution (10 mL) of CuCl**_2_**·H_2_O (200 mg, 1.173 mmol) was added dropwise an MeOH solution (10 mL) of mpmapOH (211 mg, 1.173 mmol); the color became dark blue, and the solution was stirred for 30 min at room temperature. Blue crystals of (**I**) were obtained by diffusion of diethyl ether into the dark-blue solution for several days, and were collected by filtration and washed with diethyl ether and dried in air. Yield: 247 mg (67%). FT–IR (ATR, cm^−1^): 3217, 3172, 3072, 2968, 1609, 1569, 1444, 1382, 1280, 1165, 1044, 984.

## Refinement   

Crystal data, data collection and structure refinement details are summarized in Table 2 ▸. All C-bound H atoms were placed in geometrically idealized positions and constrained to ride on their parent atoms, with C—H distances of 0.95–0.99 Å and an N—H distance of 1.0 Å. The position of the hydroxyl H atom was freely refined. All displacement parameters of H atoms *U*_iso_(H) were set to 1.2 or 1.5*U*_eq_ of their respective parent atoms.

## Supplementary Material

Crystal structure: contains datablock(s) I. DOI: 10.1107/S2056989016013773/zl2674sup1.cif

Structure factors: contains datablock(s) I. DOI: 10.1107/S2056989016013773/zl2674Isup2.hkl

CCDC reference: 1501276

Additional supporting information: crystallographic information; 3D view; checkCIF report

## Figures and Tables

**Table 1:** Hydrogen-bond geometry (Å, °)

*D*—H⋯*A*	*D*—H	H⋯*A*	*D*⋯*A*	*D*—H⋯*A*
O1—H1*O*1⋯Cl2^i^	0.84 (1)	2.19 (1)	3.0151 (10)	170 (2)
N2—H2*N*2⋯Cl1^ii^	1.00	2.40	3.3568 (11)	161

Symmetry codes: (i) 
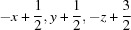
; (ii) 
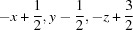
.

**Table 2:** Experimental details

Crystal data	
Chemical formula	[CuCl_2_(C_10_H_16_N_2_O)]
*M*_r_	314.69
Crystal system, space group	Monoclinic, *P*2_1_/*n*
Temperature (K)	100
*a*, *b*, *c* (Å)	8.4470 (17), 9.895 (2), 15.254 (3)
β (°)	97.13 (3)
*V* (Å^3^)	1265.1 (5)
*Z*	4
Radiation type	Synchrotron, λ = 0.610 Å
μ (mm^−1^)	1.40
Crystal size (mm)	0.12 × 0.10 × 0.09

Data collection	
Diffractometer	ADSC Q210 CCD area detector
Absorption correction	Empirical (using intensity measurements) (*HKL-3000* *SCALEPACK*; Otwinowski & Minor, 1997 ▸)
*T*_min_, *T*_max_	0.809, 0.887
No. of measured, independent and observed [*I* > 2σ(*I*)] reflections	11018, 3674, 3556
*R*_int_	0.031
(sin θ/λ)_max_ (Å^−1^)	0.706

Refinement	
*R*[*F* ^2^ > 2σ(*F* ^2^)], *wR*(*F* ^2^), *S*	0.022, 0.061, 1.06
No. of reflections	3674
No. of parameters	148
H-atom treatment	H atoms treated by a mixture of independent and constrained refinement
Δρ_max_, Δρ_min_ (e Å^−3^)	0.51, −0.90

Computer programs: *PAL BL2D-SMDC* (Shin *et al.*, 2016 ▸), *HKL3000sm* (Otwinowski & Minor, 1997 ▸), *SHELXT2014* (Sheldrick, 2015*a* ▸), *SHELXL2014* (Sheldrick, 2015*b* ▸), *DIAMOND* (Putz & Brandenburg, 2014 ▸) and *publCIF* (Westrip, 2010 ▸).

## supplementary crystallographic information

### Crystal data

**Table:**

[CuCl_2_(C_10_H_16_N_2_O)]	*F*(000) = 644
*M**_r_* = 314.69	*D*_x_ = 1.652 Mg m^−^^3^
Monoclinic, *P*2_1_/*n*	Synchrotron radiation, λ = 0.610 Å
*a* = 8.4470 (17) Å	Cell parameters from 24265 reflections
*b* = 9.895 (2) Å	θ = 0.4–33.7°
*c* = 15.254 (3) Å	µ = 1.40 mm^−^^1^
β = 97.13 (3)°	*T* = 100 K
*V* = 1265.1 (5) Å^3^	Block, blue
*Z* = 4	0.12 × 0.10 × 0.09 mm

### Data collection

**Table:**

ADSC Q210 CCD area detector diffractometer	3556 reflections with *I* > 2σ(*I*)
Radiation source: PLSII 2D bending magnet	*R*_int_ = 0.031
ω scan	θ_max_ = 25.5°, θ_min_ = 2.9°
Absorption correction: empirical (using intensity measurements) (HKL3000 Scalepack; Otwinowski & Minor, 1997)	*h* = −11→11
*T*_min_ = 0.809, *T*_max_ = 0.887	*k* = −13→13
11018 measured reflections	*l* = −21→21
3674 independent reflections	

### Refinement

**Table:**

Refinement on *F*^2^	0 restraints
Least-squares matrix: full	Hydrogen site location: inferred from neighbouring sites
*R*[*F*^2^ > 2σ(*F*^2^)] = 0.022	H atoms treated by a mixture of independent and constrained refinement
*wR*(*F*^2^) = 0.061	*w* = 1/[σ^2^(*F*_o_^2^) + (0.0336*P*)^2^ + 0.6535*P*] where *P* = (*F*_o_^2^ + 2*F*_c_^2^)/3
*S* = 1.06	(Δ/σ)_max_ = 0.004
3674 reflections	Δρ_max_ = 0.51 e Å^−^^3^
148 parameters	Δρ_min_ = −0.90 e Å^−^^3^

### Special details

**Table:**

Geometry. All esds (except the esd in the dihedral angle between two l.s. planes) are estimated using the full covariance matrix. The cell esds are taken into account individually in the estimation of esds in distances, angles and torsion angles; correlations between esds in cell parameters are only used when they are defined by crystal symmetry. An approximate (isotropic) treatment of cell esds is used for estimating esds involving l.s. planes.

### Fractional atomic coordinates and isotropic or equivalent isotropic displacement parameters (Å^2^)

**Table:**

	*x*	*y*	*z*	*U*_iso_*/*U*_eq_	
Cu1	0.28124 (2)	0.60310 (2)	0.68527 (2)	0.00421 (5)	
Cl1	0.19986 (4)	0.77197 (3)	0.59216 (2)	0.01365 (7)	
Cl2	0.02903 (3)	0.50366 (3)	0.72731 (2)	0.01123 (7)	
O1	0.31493 (9)	0.73256 (8)	0.79008 (5)	0.00605 (14)	
H1O1	0.3621 (18)	0.8042 (12)	0.7791 (11)	0.007*	
N1	0.32349 (11)	0.46940 (9)	0.59369 (6)	0.00555 (16)	
N2	0.43588 (10)	0.48634 (9)	0.76378 (6)	0.00424 (15)	
H2N2	0.3706	0.4292	0.7998	0.005*	
C1	0.24193 (13)	0.46123 (12)	0.51215 (7)	0.00921 (19)	
H1	0.1599	0.5252	0.4952	0.011*	
C2	0.27367 (14)	0.36289 (13)	0.45236 (7)	0.0113 (2)	
H2	0.2134	0.3580	0.3956	0.014*	
C3	0.39622 (14)	0.27116 (13)	0.47736 (8)	0.0121 (2)	
H3	0.4234	0.2046	0.4369	0.015*	
C4	0.47810 (13)	0.27800 (12)	0.56195 (8)	0.0103 (2)	
H4	0.5602	0.2149	0.5806	0.012*	
C5	0.43811 (12)	0.37864 (11)	0.61898 (7)	0.00587 (18)	
C6	0.52329 (13)	0.39249 (11)	0.71132 (7)	0.00744 (19)	
H6A	0.5317	0.3028	0.7402	0.009*	
H6B	0.6326	0.4270	0.7088	0.009*	
C7	0.53496 (12)	0.57791 (11)	0.82728 (7)	0.00457 (17)	
C8	0.64485 (12)	0.66575 (11)	0.77880 (7)	0.00812 (18)	
H8A	0.7355	0.6116	0.7647	0.012*	
H8B	0.6840	0.7418	0.8166	0.012*	
H8C	0.5856	0.7002	0.7241	0.012*	
C9	0.63253 (14)	0.49822 (12)	0.90080 (7)	0.00968 (19)	
H9A	0.5613	0.4402	0.9303	0.015*	
H9B	0.6878	0.5611	0.9438	0.015*	
H9C	0.7111	0.4422	0.8755	0.015*	
C10	0.40927 (12)	0.66639 (11)	0.86317 (7)	0.00648 (18)	
H10A	0.3397	0.6100	0.8960	0.008*	
H10B	0.4620	0.7348	0.9042	0.008*	

### Atomic displacement parameters (Å^2^)

**Table:**

	*U*^11^	*U*^22^	*U*^33^	*U*^12^	*U*^13^	*U*^23^
Cu1	0.00445 (7)	0.00330 (8)	0.00458 (7)	0.00238 (4)	−0.00066 (5)	−0.00020 (4)
Cl1	0.02298 (14)	0.00915 (13)	0.00860 (12)	0.00964 (10)	0.00104 (10)	0.00304 (9)
Cl2	0.00569 (11)	0.00647 (12)	0.02190 (14)	0.00083 (8)	0.00314 (9)	−0.00072 (9)
O1	0.0060 (3)	0.0038 (3)	0.0080 (3)	0.0009 (2)	−0.0002 (3)	−0.0005 (3)
N1	0.0066 (4)	0.0049 (4)	0.0051 (4)	0.0009 (3)	0.0002 (3)	−0.0002 (3)
N2	0.0039 (3)	0.0036 (4)	0.0049 (4)	0.0009 (3)	−0.0006 (3)	−0.0007 (3)
C1	0.0101 (4)	0.0106 (5)	0.0063 (4)	0.0002 (4)	−0.0013 (3)	0.0003 (4)
C2	0.0132 (5)	0.0143 (5)	0.0063 (4)	−0.0032 (4)	0.0002 (4)	−0.0024 (4)
C3	0.0117 (5)	0.0143 (5)	0.0107 (5)	−0.0017 (4)	0.0025 (4)	−0.0074 (4)
C4	0.0077 (4)	0.0095 (5)	0.0133 (5)	0.0017 (4)	0.0002 (4)	−0.0067 (4)
C5	0.0048 (4)	0.0057 (4)	0.0070 (4)	−0.0004 (3)	0.0005 (3)	−0.0020 (3)
C6	0.0073 (4)	0.0061 (5)	0.0082 (4)	0.0040 (3)	−0.0017 (4)	−0.0034 (3)
C7	0.0044 (4)	0.0045 (4)	0.0044 (4)	0.0000 (3)	−0.0009 (3)	−0.0010 (3)
C8	0.0057 (4)	0.0071 (4)	0.0118 (5)	−0.0012 (3)	0.0024 (3)	0.0006 (4)
C9	0.0106 (5)	0.0094 (5)	0.0076 (4)	0.0016 (4)	−0.0044 (4)	0.0005 (4)
C10	0.0068 (4)	0.0074 (4)	0.0053 (4)	0.0018 (3)	0.0007 (3)	−0.0011 (3)

### Geometric parameters (Å, º)

**Table:**

Cu1—N1	1.9881 (10)	C3—H3	0.9500
Cu1—N2	2.0217 (10)	C4—C5	1.3913 (15)
Cu1—O1	2.0409 (9)	C4—H4	0.9500
Cu1—Cl1	2.2448 (5)	C5—C6	1.5063 (16)
Cu1—Cl2	2.5014 (6)	C6—H6A	0.9900
O1—C10	1.4444 (13)	C6—H6B	0.9900
O1—H1O1	0.840 (9)	C7—C9	1.5262 (15)
N1—C5	1.3417 (14)	C7—C8	1.5278 (15)
N1—C1	1.3474 (14)	C7—C10	1.5289 (14)
N2—C6	1.4814 (13)	C8—H8A	0.9800
N2—C7	1.5028 (14)	C8—H8B	0.9800
N2—H2N2	1.0000	C8—H8C	0.9800
C1—C2	1.3825 (16)	C9—H9A	0.9800
C1—H1	0.9500	C9—H9B	0.9800
C2—C3	1.3938 (17)	C9—H9C	0.9800
C2—H2	0.9500	C10—H10A	0.9900
C3—C4	1.3880 (16)	C10—H10B	0.9900
			
N1—Cu1—N2	82.92 (4)	N1—C5—C4	121.46 (10)
N1—Cu1—O1	161.51 (4)	N1—C5—C6	116.87 (9)
N2—Cu1—O1	82.97 (4)	C4—C5—C6	121.67 (10)
N1—Cu1—Cl1	96.80 (3)	N2—C6—C5	110.48 (9)
N2—Cu1—Cl1	157.64 (3)	N2—C6—H6A	109.6
O1—Cu1—Cl1	91.74 (3)	C5—C6—H6A	109.6
N1—Cu1—Cl2	98.69 (3)	N2—C6—H6B	109.6
N2—Cu1—Cl2	97.56 (3)	C5—C6—H6B	109.6
O1—Cu1—Cl2	94.96 (3)	H6A—C6—H6B	108.1
Cl1—Cu1—Cl2	104.55 (2)	N2—C7—C9	111.65 (9)
C10—O1—Cu1	109.28 (6)	N2—C7—C8	110.75 (8)
C10—O1—H1O1	107.8 (12)	C9—C7—C8	110.15 (9)
Cu1—O1—H1O1	113.4 (12)	N2—C7—C10	102.72 (8)
C5—N1—C1	119.56 (10)	C9—C7—C10	111.62 (9)
C5—N1—Cu1	115.43 (7)	C8—C7—C10	109.76 (9)
C1—N1—Cu1	124.94 (8)	C7—C8—H8A	109.5
C6—N2—C7	116.81 (8)	C7—C8—H8B	109.5
C6—N2—Cu1	111.52 (7)	H8A—C8—H8B	109.5
C7—N2—Cu1	107.72 (7)	C7—C8—H8C	109.5
C6—N2—H2N2	106.7	H8A—C8—H8C	109.5
C7—N2—H2N2	106.7	H8B—C8—H8C	109.5
Cu1—N2—H2N2	106.7	C7—C9—H9A	109.5
N1—C1—C2	122.17 (11)	C7—C9—H9B	109.5
N1—C1—H1	118.9	H9A—C9—H9B	109.5
C2—C1—H1	118.9	C7—C9—H9C	109.5
C1—C2—C3	118.43 (11)	H9A—C9—H9C	109.5
C1—C2—H2	120.8	H9B—C9—H9C	109.5
C3—C2—H2	120.8	O1—C10—C7	108.92 (8)
C4—C3—C2	119.35 (11)	O1—C10—H10A	109.9
C4—C3—H3	120.3	C7—C10—H10A	109.9
C2—C3—H3	120.3	O1—C10—H10B	109.9
C3—C4—C5	118.99 (11)	C7—C10—H10B	109.9
C3—C4—H4	120.5	H10A—C10—H10B	108.3
C5—C4—H4	120.5		
			
C5—N1—C1—C2	−0.93 (17)	N1—C5—C6—N2	13.48 (13)
Cu1—N1—C1—C2	−177.79 (9)	C4—C5—C6—N2	−167.61 (10)
N1—C1—C2—C3	−1.17 (18)	C6—N2—C7—C9	−64.80 (12)
C1—C2—C3—C4	2.37 (18)	Cu1—N2—C7—C9	168.82 (7)
C2—C3—C4—C5	−1.55 (18)	C6—N2—C7—C8	58.33 (12)
C1—N1—C5—C4	1.81 (16)	Cu1—N2—C7—C8	−68.04 (9)
Cu1—N1—C5—C4	178.96 (9)	C6—N2—C7—C10	175.47 (8)
C1—N1—C5—C6	−179.28 (10)	Cu1—N2—C7—C10	49.09 (8)
Cu1—N1—C5—C6	−2.13 (12)	Cu1—O1—C10—C7	37.17 (9)
C3—C4—C5—N1	−0.56 (17)	N2—C7—C10—O1	−56.79 (10)
C3—C4—C5—C6	−179.42 (11)	C9—C7—C10—O1	−176.54 (8)
C7—N2—C6—C5	−142.45 (9)	C8—C7—C10—O1	61.04 (11)
Cu1—N2—C6—C5	−17.98 (10)		

### Hydrogen-bond geometry (Å, º)

**Table:**

*D*—H···*A*	*D*—H	H···*A*	*D*···*A*	*D*—H···*A*
O1—H1*O*1···Cl2^i^	0.84 (1)	2.19 (1)	3.0151 (10)	170 (2)
N2—H2*N*2···Cl1^ii^	1.00	2.40	3.3568 (11)	161

Symmetry codes: (i) −*x*+1/2, *y*+1/2, −*z*+3/2; (ii) −*x*+1/2, *y*−1/2, −*z*+3/2.
